# Multidrug-Resistant Bacteria in Immunocompromised Patients

**DOI:** 10.3390/ph17091151

**Published:** 2024-08-30

**Authors:** Alexandru Duhaniuc, Diana Păduraru, Eduard-Vasile Nastase, Felicia Trofin, Luminița-Smaranda Iancu, Cristina-Mihaela Sima, Olivia-Simona Dorneanu

**Affiliations:** 1 Department of Preventive Medicine and Interdisciplinarity—Microbiology, University of Medicine and Pharmacy “Grigore T. Popa”, 700115 Iasi, Romania; alexandru.duhaniuc@umfiasi.ro (A.D.); luminita.iancu@umfiasi.ro (L.-S.I.); cristina.sima@umfiasi.ro (C.-M.S.); olivia.dorneanu@umfiasi.ro (O.-S.D.); 2 National Institute of Public Health—Regional Center of Public Health, 700465 Iasi, Romania; 3 “Dr. C.I. Parhon” Clinical Hospital, 700503 Iasi, Romania; diana_paduraru@email.umfiasi.ro; 4 Department of Internal Medicine II—Infectious Diseases, University of Medicine and Pharmacy “Grigore T. Popa”, 700115 Iasi, Romania; 5 Clinical Hospital of Infectious Diseases “Sf. Parascheva”, 700116 Iasi, Romania

**Keywords:** multidrug-resistant bacteria, immunocompromised patients, colonization, infection

## Abstract

The increasing incidence of antibiotic resistance in bacteria is a major problem in terms of therapeutic options, especially in immunocompromised patients, such as patients from intensive care units (ICUs), HIV-positive patients, patients with malignancies or transplant patients. Commensal bacteria, especially anaerobes, serve to maintain microbial stability by preventing overpopulation with pathogenic bacteria. In immunocompromised patients, microbiota imbalance caused by antibiotic therapy and decreased host immunity favors intestinal overpopulation with pathogenic species, leading to increased bacterial translocation and susceptibility to systemic infections. Infections with multidrug-resistant (MDR) bacteria pose major challenges to the establishment of appropriate treatment and lead to increased mortality. Asymptomatic colonization with MDR bacteria usually precedes infection and tends to persist for long periods of time, and in immunocompromised patients, colonization with MDR bacteria is a risk factor for systemic infections. This review aims to assess the relation between colonization and infection with MDR bacteria in immunocompromised patients such as ICU patients, HIV-positive patients and cancer patients and to identify the prevalence and patterns of MDR bacterial colonization and infection in this category of patients.

## 1. Introduction

Multidrug-resistant (MDR) microorganisms are defined as microorganisms that exhibit resistance to at least three classes of antibiotics, and the most commonly encountered are extended-spectrum beta-lactamase (ESBL)-producing *Enterobacterales*, carbapenem-resistant *Enterobacterales* (CRE), carbapenem-resistant *Pseudomonas aeruginosa* and *Acinetobacter baumannii*, methicillin-resistant *Staphylococcus aureus* (MRSA) and vancomycin-resistant *Enterococcus* spp. (VRE) [1]. Antibiotic resistance is currently one of the main threats to global health, requiring urgent action to prevent its spread. Antibiotic resistance has been recognized by the World Health Organization (WHO) as a major threat to public health, and the WHO has prioritized pathogens based on several criteria, such as the mortality of infections caused by them, transmissibility, treatability and ways to prevent infections caused by them in healthcare facilities and in the community. The MDR bacteria included in this list were classified into three priority groups (critical, high and medium) according to antibiotic resistance, and this classification was made in order to prioritize and stimulate the development of new antimicrobial agents for these pathogens. The critical-priority group included carbapenem-resistant *Enterobacterales*, third-generation cephalosporin-resistant *Enterobacterales* and carbapenem-resistant *A. baumannii*. The high-priority group included vancomycin-resistant *Enterococcus faecium*, carbapenem-resistant *P. aeruginosa*, methicillin-resistant *S. aureus*, fluoroquinolone-resistant *Salmonella* Typhi and non-typhoidal *Salmonella* and *Shigella* spp., and the medium-priority group included macrolide-resistant *Streptococcus pneumoniae* and ampicillin-resistant *Haemophilus influenzae* [2]. According to the ECDC, a significant increase in the number of carbapenem-resistant strains of *Escherichia coli* and *Klebsiella pneumoniae* as well as vancomycin-resistant strains of *E. faecium* has been reported in Europe from 2016 to 2020. In 2020, more than half of the *E. coli* strains and more than a third of the *K. pneumoniae* strains were resistant to at least one class of antibiotics, and combined resistance to several classes of antibiotics was common [3]. In 2021, 53.1% of *E. coli* strains, 34.3% of *K. pneumoniae* strains and 18.7% of *P. aeruginosa* strains were reported to be resistant to at least one class of antibiotics. Carbapenem resistance remained rare for *E. coli* strains, but a quarter of European countries reported carbapenem resistance rates above 10% for *K. pneumoniae* strains. This percentage is increasing, with a 20% increase in carbapenem-resistant *K. pneumoniae* strains reported in 2021 compared to the previous year [4]. In 2022, the most frequently reported bacteria were *E. coli* (39.2%), followed by *S. aureus* (22.1%), *K. pneumoniae* (12.3%), *E. faecalis* (8.2%), *P. aeruginosa* (6.1%), *E. faecium* (5.9%) and *Acinetobacter* spp. (2.5%). The incidence of bloodstream infections with MRSA and *E. coli* resistant to third-generation cephalosporins decreased between 2019 and 2022, but the incidence of carbapenem-resistant *K. pneumoniae* increased by almost 50%. The most recent concern in Europe regarding antimicrobial resistance is the continuous increase in carbapenem-resistant *K. pneumoniae* and vancomycin-resistant *E. faecium* [5]. Multidrug resistance is not the same across Europe, with countries in Southern and Eastern Europe showing higher percentages of MDR bacteria compared to those in Northern and Western Europe [3,4,5].

Because *Enterobacterales* are commensals of the intestinal microbiota, infections with MDR *Enterobacterales* may occur due to prior gut colonization, especially in hospital settings where carriers of such bacteria can easily transmit them to other people in close contact with them [6,7]. In hospital settings, people colonized with MDR bacteria should be rapidly identified in order to prevent infections and contain the spread of these bacteria [8]. In immunocompromised patients, such as patients from ICUs, HIV-positive patients, patients with cancer or diabetes and transplant patients, prolonged antibiotic prophylaxis, invasive procedures and the frequent contact with the healthcare system are known factors contributing to the colonization with MDR bacteria. This category of patients has a higher prevalence of infections with MDR bacteria, especially Gram-negative bacilli (GNB), which are difficult to treat, and leads to increased mortality in these patients [9]. Asymptomatic colonization with MDR bacteria usually precedes infection and tends to persist for long periods of time. In this case, treatment can be administered to eradicate or reduce the carriage of MDR bacteria during periods when the patient is most vulnerable [10]. However, the 2019 ESCMID-EUCIC clinical guidelines do not recommend routine decolonization, as the results of studies using non-absorbable antibiotics, or other microbiota-altering methods such as probiotics or fecal microbiota transplantation, for MDR bacteria colonizing the intestinal tract have been largely disappointing, with mild suppression of carriage but rapid reappearance after cessation of treatment. The guidelines suggest, however, that prospective clinical trials in immunocompromised patients should be conducted, based on the idea that temporary colonization suppression may be beneficial in this category of patients [11].

Distinguishing between infection and colonization by MDR bacteria in these patients is critical for several reasons. Firstly, immunocompromised patients are at a heightened risk of developing severe infections due to their weakened immune systems. Understanding whether the presence of MDR bacteria indicates colonization or an active infection can guide appropriate clinical management, including the necessity and choice of antimicrobial therapy. Secondly, differentiating between colonization and infection is vital for effective infection control measures. Misclassification can lead to unnecessary treatment, which contributes to antibiotic resistance, or inadequate precautions, which can result in the spread of MDR bacteria within healthcare settings. Thirdly, the outcomes and prognosis for immunocompromised patients can vary significantly depending on whether they are merely colonized by MDR bacteria or actively infected. Accurate differentiation is essential for predicting patient outcomes and tailoring individualized care plans. Additionally, comprehensive data on the epidemiology of MDR bacteria in immunocompromised patients can inform public health policies and research priorities. Such data can aid in the development of new guidelines for the prevention, early detection and management of MDR bacterial infections in this high-risk group. Moreover, healthcare resources, including isolation measures and antimicrobial stewardship efforts, must be judiciously allocated. Clear differentiation helps optimize resource use, ensuring that patients with active infections receive necessary care without overburdening healthcare systems. Given these critical factors, a comprehensive review of the topic is warranted. So, this review is aimed to assess the clinical implications of MDR bacterial colonization versus infection, to compile and synthesize epidemiological data on the prevalence and patterns of MDR bacterial colonization and infection in immunocompromised patients across various healthcare settings and regions and to identify gaps in the existing research and knowledge, proposing areas for further study to improve the understanding and management of MDR bacteria in immunocompromised populations.

## 2. Materials and Methods

**Figure 1:**
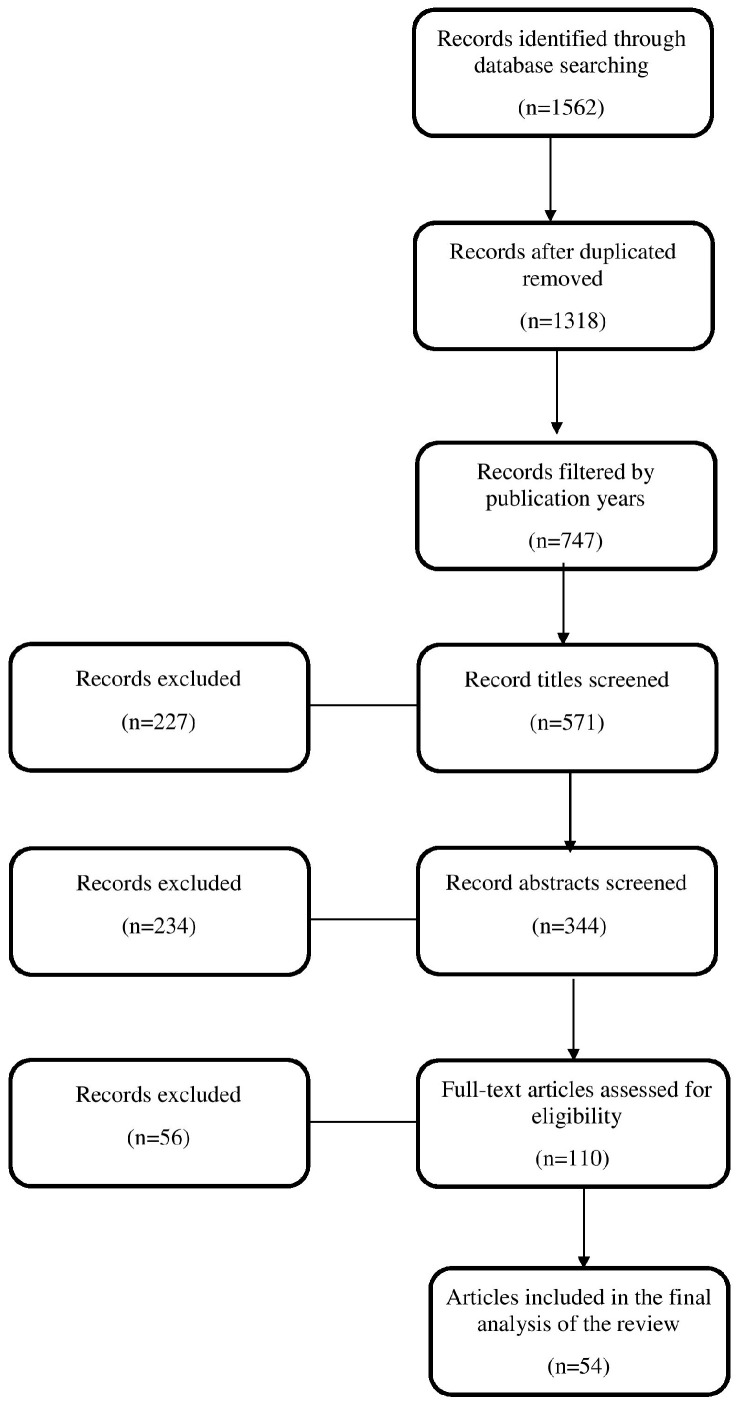
The selection process of the articles.

This review encompasses 54 studies. The initial records were identified through a database search using the terms “multidrug resistant bacteria”, “colonization”, “infection”, “immunocompromised patients”, “ICU”, “HIV” and “cancer”. Methodical searches were conducted using established databases, including PubMed, Embase, Scopus and Google Scholar. Following the initial database searches, a comprehensive screening process was undertaken, starting with the removal of duplicate records. After screening the titles, some articles were excluded due to a misalignment between their titles and the research objectives of this review. During the abstract examination, various articles were excluded based on factors, such as topical relevance, publication date, accessibility constraints and overall pertinence to the research inquiry. A thorough examination of the full texts led to the exclusion of additional articles due to relevance issues, methodological incongruity, scope misalignment, quality and credibility concerns or language barriers. The accompanying flowchart (Figure 1) illustrates the sequential progression of information through the review process, depicting the number of records identified, included and excluded.

The selection and curation of articles for inclusion in our review adhered to rigorous criteria, ensuring alignment with our central research question: “Which are the relationships between colonization and infection with multidrug-resistant bacteria in immunocompromised patients?”. The criteria included adherence to the research objectives, consideration of the publication year (2019–2024), categorization of scientific research and an assessment of the quality of findings presentation. The searches were meticulously structured with relevant keywords and phrases and further refined using Boolean operators such as “and” and “or” to enhance search precision. The selected studies underwent a qualitative analysis, scrutinizing adherence to the principles of scholarly composition, clarity, brevity, citation frequency, sample size, provision of pertinent data, articulation of results and formulation of conclusions. These aspects were integrated into the final narrative synthesis. Additionally, supplementary references meeting the defined criteria were identified through a manual search of the reference lists of the retrieved articles.

## 3. Colonization and Infection with MDR Bacteria in ICU Patients

In ICU settings, most infections are related to MDR bacteria, and the prevalence of MDR bacteria in the ICU is determined by the admission of patients colonized, as well as the number of patients that acquire MDR bacteria during their stay in the ICU [12,13,14]. A series of frequent invasive medical procedures such as intubation, mechanical ventilation and vascular access leave the ICU patients more vulnerable to infections with GNB [15]. The ICU is often considered the main source of MDR GNB, given the frequent and incorrect use of broad-spectrum antibiotics that lead to the emergence of drug-resistant strains, and the bacterial exchange of resistance genes, such as plasmid-encoded β-lactamases, aminoglycosides-modifying enzymes and quinolone resistance genes [16,17]. Therefore, the ICU is an environment where there is a high risk of amplifying the prevalence of MDR bacteria if infection control measures are not strictly applied [14]. Prior to the pandemic, several studies showed that ICU-acquired colonization with MDR bacteria was associated with a longer ICU stay, while ICU-acquired infections with MDR bacteria were associated to a higher mortality [12,18,19]. The successful spread of MDR bacteria in both the community and the hospital led to increased rates of colonization with ESBL-producing *Enterobacterales* in the ICU worldwide. Patients who are colonized with MDR bacteria serve as potential reservoirs for transmission, so early detection of MDR carriers should be performed in order to help prevent disease and dissemination [20].

Several studies from different regions of the world conducted on ICU patients focused on the colonization with MDR bacteria upon admission to the ICU, while others focused on the bacterial infections of ICU patients and the susceptibility profile of bacteria to antibiotics (Table 1). A study from Spain regarding colonization with MDR bacteria showed that there was a predominance of MDR GNB upon ICU admission. The most frequently isolated species were *E. coli* (57%) and *K. pneumoniae* (16%), followed by *Citrobacter freundii* (7%), *Enterobacter cloacae* (6%) and *Klebsiella aerogenes* (5%). Regarding antimicrobial resistance mechanisms, the most common were ESBL-producing *E. coli* and other ESBL producers (76%) and derepressed ampC producers (15%). This study also showed that liver cirrhosis, previous MDR-GNB carriage, digestive surgery in the last year and length of hospital stay were independent risk factors for MDR-GNB carriage upon ICU admission [21]. Another study from Turkey that focused on colonization with VRE and *K. pneumoniae* in 230 ICU patients showed that 36 patients (16%) were colonized with VRE and 7 patients (3%) were colonized with *K. pneumoniae*. The majority of VRE strains (81%) were represented by *E. faecium* and the most common genotype was *VanA*; meanwhile, all strains of *K. pneumoniae* were positive for OXA-48, while some of them were also identified as positive for NDM [22]. A study from Greece on the de-escalation of empirical antimicrobial therapy in 262 septic patients with documented infections in ICUs with a high prevalence of antimicrobial resistance showed that de-escalation was not feasible in approximately one-third of patients due to the identification of antibiotic-resistant pathogens, but when antimicrobial de-escalation was implemented, 28-day mortality was lower and both ICU and hospital mortality also decreased. The most frequently isolated pathogens were *A. baumannii* (n = 93; 35.5%), *K. pneumoniae* (n = 54; 20.6%), *Enterococcus* spp. (n = 34; 13%), *P. aeruginosa* (n = 30; 11.5%), *S. aureus* (n = 21; 8%) and *E. coli* (n = 18; 6.9%). Antibiotic-resistant pathogens accounted for 62.9% of the total, classified as MDR (12.5%), extensive drug resistant (XDR) (49.2%), and pandrug resistant (PDR) (1.2%) [23]. A study from Italy on 420 neurocritical patients admitted to the ICU showed that 167 patients had Gram-negative colonization, while 32 patients had Gram-positive colonization, with 11 patients showing overlap between the two. In the Gram-negative group, 73 patients (43.71%) developed colonization by MDR pathogens and 53 patients (31.73%) by XDR Gram-negative pathogens. Among the patients colonized by MDR pathogens, 33 (45.20%) out of 73 developed sepsis, whereas 17 (32.07%) out of 53 patients with XDR colonization were diagnosed with an infection. In this study, ICU length of stay was identified as the strongest predictor for colonization by both MDR and XDR Gram-negative pathogens. Additionally, the use of external ventricular drainage and intracerebral pressure-monitoring catheters were identified as risk factors for XDR colonization, so this indicates that early removal of these catheters should be considered as soon as it is clinically appropriate [24]. Another study from Italy conducted on 54 patients with severe trauma admitted to the ICU showed that 28 patients were colonized by carbapenem-resistant GNB. Of these, 7 patients (12.96%) were already colonized at ICU admission, while 21 patients (38.89%) developed new colonization during their ICU stay. All colonization cases involved carbapenem-resistant *A. baumannii* and/or carbapenem-resistant *K. pneumoniae*. This study showed that a longer ICU stay (14.81 ± 9.1 days for non-colonized patients vs. 38.19 ± 27.9 days for colonized patients; *p* = 0.001) and extended periods of mechanical ventilation (8.46 ± 7.67 days for non-colonized patients vs. 22.19 ± 15.09 days for colonized patients; *p* < 0.001) were risk factors associated with colonization, and they also showed that there was a strong statistical association between prior colonization and the subsequent development of infection (*p* < 0.001) [25]. In Romania, a study on 253 ICU patients showed that 53 patients (20.9%) were colonized upon admission to the ICU. A total of 37 patients (68.8%) were colonized with one bacterial strain and the most frequently isolated strains were *E. coli*, *Klebsiella* spp., *S. aureus* and *E. faecium*, while 13 patients (24.5%) were colonized with two different bacterial strains, with the most common association being *Klebsiella* spp. and *E. faecium*. Only three patients (5.7%) were colonized with three different bacterial strains. The predominant antimicrobial resistance mechanism detected was the production of ESBL (75.5%), followed by the production of carbapenemases (32.1%) [26].

A study from China that focused on colonization with *K. pneumoniae* showed that 28% of ICU patients (68 of 243 patients) were colonized with carbapenem-resistant *K. pneumoniae,* and those that were colonized with carbapenem-susceptible *K. pneumoniae* upon admission were more likely to become colonized with carbapenem-resistant *K. pneumoniae* during their stay in the ICU, compared to those that were not colonized with *K. pneumoniae*. Also, the incidence of subsequent infection in people colonized with carbapenem-susceptible *K. pneumoniae* (32.3%) and carbapenem-resistant *K. pneumoniae* (45.9%) was higher than in those without *K. pneumoniae* colonization (8.6%) [27]. A study from Korea on 77 ICU patients colonized with CRE showed that 51 patients (66.2%) were colonized upon ICU admission, while 26 patients (33.8%) acquired CRE during their ICU stay. The most frequently isolated CRE was *K. pneumoniae* in 60 patients and *E. coli* in 14 patients. KPC-producing bacteria were the most commonly isolated, found in 69 patients (89.6%), followed by NDM-producing bacteria found in 5 patients, OXA-48-producing bacteria in 1 patient, and both NDM- and OXA-48-producing bacteria in 2 patients. They also showed that the acquisition of CRE was significantly linked to ICU stay, open wounds, the presence of catheters or tubes and antibiotic treatment [28]. In Saudi Arabia, a study showed that *K. pneumoniae* (21%), *P. aeruginosa* (11.8%) and *S. aureus* (13.2%) were the most common bacteria causing infections in ICU patients. The majority of GNB (85.1%) were MDR, 28.6% were XDR and 4.6% were PDR. Among the frequently isolated GNB, *P. aeruginosa* (95.2%), *Proteus mirabilis* (86.4%), *K. pneumoniae* (83.3%) and *E. coli* (79.4%) were MDR. All isolates of *A. baumannii* were MDR and all isolates of *Burkholderia cepacia* were PDR [29].

In a study conducted in a neonatal ICU from Tanzania, 43% of neonates (86 of 200 neonates) were colonized with GNB resistant to third-generation cephalosporins, with *K. pneumoniae*, *Acinetobacter* spp., *E. coli* and *C. freundii* being frequently isolated. These GNB were commonly isolated from both blood and rectal swabs suggesting that rectal colonization may be the source of bacteremia. More than 90% of GNB isolated from blood and rectal swabs were MDR [30]. Another study from Tanzania on 198 ICU patients focused on colonization with ESBL-producing *Enterobacterales* and MRSA. They showed that 54.54% of patients (108 out of 198) had fecal carriage of ESBL-producing *Enterobacterales* upon ICU admission, and 9.32% of patients (15 out of 161) had nasal carriage of MRSA. They also suggested that risk factors such as hospitalization for two or more days before ICU admission (*p* > 0.05) and antibiotic use prior to ICU admission (*p* = 0.285) may have an impact on intestinal colonization with ESBL-producing *Enterobacterales*, though these associations were not statistically significant. However, they showed that previous hospitalization in the last six months could be protective against fecal carriage of ESBL-producing *Enterobacterales* (*p* = 0.037). On the other hand, nasal MRSA colonization was more likely among patients who had received antibiotics prior to ICU admission, but this association was not statistically significant (*p* = 0.491) [31]. A study from Kenya regarding bacterial infections of 162 ICU patients showed that 90 patients (55.6%) had bacterial infections with GNB, the most common infections being urinary tract infections, wound infections and lower respiratory tract infections. The most common isolates were *E. coli* (33.3%), *K. pneumoniae* (31.1%) and *P. aeruginosa* (14.4%). In this study, 92% of isolates were MDR, with *E. coli* (90%)*, K. pneumoniae* (89.3%) and *P. aeruginosa* (100%) as the most frequent MDR isolates. *Enterobacterales* showed resistance to third-generation cephalosporins, ranging from 50% to 100%, and to carbapenems, ranging from 46% to 54% for *K. pneumoniae* and 10% to 27% for *E. coli*. The highest carbapenem resistance was observed in non-fermenting GNB, such as *A. baumannii* and *P. aeruginosa*, ranging from 60% to 100%. Colistin resistance was high in *A. baumannii* and *P. aeruginosa* (60 to 92%) and lower in *E. coli* and *K. pneumoniae* (17 to 46%) [32]. 

In a Brazilian ICU, 9.5% were positive for MDR bacteria and the most frequently isolated species were *A. baumannii* (25.4%), *Acinetobacter* spp. (14.3%), *K. pneumoniae* (13.2%), *P. aeruginosa* (9.6%) and *S. aureus* (8.7%). All strains of *Acinetobacter* and *P. aeruginosa* were resistant to carbapenems, all strains of *S. aureus* were methicillin-resistant and *K. pneumoniae* strains were 76% ESBL and 22% KPC [33]. Another study from Brazil on 267 ICU patients diagnosed with lower respiratory tract infections showed that 146 patients (62%) had at least one MDR isolate. The most frequently isolated MDR bacteria were carbapenem-resistant *A. baumannii* and *P. aeruginosa*, carbapenem-resistant *K. pneumoniae*, MRSA and other CRE. Patients infected with MDR strains experienced worse outcomes compared to those infected with susceptible strains, including longer durations of mechanical ventilation (18 days vs. 12 days; *p* < 0.001), extended ICU stays (23 days vs. 16 days; *p* < 0.001) and higher mortality rates (73% vs. 53%; *p* < 0.001) [34]. A study from Mexico that assessed the antimicrobial resistance of bacteria isolated from infections in patients from neonatal, pediatric and adult ICUs showed that the most common GNB isolated from infections were *P. aeruginosa* (30.17%), *K. pneumoniae* (20.12%) and *E. coli* (16.05%). In neonatal and pediatric ICUs, the most common ones were *P. aeruginosa* and *K. pneumoniae*, while in adult ICUs, the most common ones were *E. coli*, *K. pneumoniae*, *A. baumannii* and *P. aeruginosa*. The highest percentage of MDR was present for *E. coli* (91.57%), *A. baumannii* (86.79%) and *K. pneumoniae* (83.65%), and overall, 71.65% of the total GNB was MDR [35]. 

A study from the USA conducted on 600 ICU patients with cirrhosis showed that at the time of ICU admission, 200 patients (33%) were colonized with at least one MDR bacteria: 169 patients (28%) were colonized with VRE, 34 patients (6%) were colonized with MRSA and 16 patients (3%) were colonized with ESBL-producing *Enterobacterales*. During the ICU stay, infections occurred in 347 patients (58%), with 147 of these cases (42%) being ICU-acquired, and 69 patients (12%) developed infections with MDR bacteria. In this study, MDR colonization was significantly associated with MDR infection (*p* < 0.001), and both MDR colonization and MDR infection were linked to a higher number and longer duration of antibiotic use (*p* < 0.001). Moreover, MDR colonization and MDR infection were independently associated with reduced transplant-free survival and a poorer prognosis among critically ill cirrhosis patients [36]. A study by Prado et al. assessed MDR colonization and infection in two series of critically ill patients: the Barcelona cohort, which included 486 patients (129 with cirrhosis and 357 without), and the Frankfurt cohort, which included 421 patients with cirrhosis. In the Barcelona cohort, 159 patients (32.7%) were colonized by MDR bacteria, with 102 (64.2%) at admission and 57 (35.8%) during follow-up. Patients with cirrhosis had higher rates of rectal colonization at admission compared to those without cirrhosis (28.7% vs. 18.2%; *p* = 0.01), though the rates were similar during the ICU stay. The most frequently isolated MDR bacteria in both groups was ESBL-producing *Enterobacterales*. MDR colonization independently increased the risk of MDR infection at admission and during follow-up, with a significantly higher risk of new infection by the colonizing strain for both patients with and without cirrhosis. In the Frankfurt cohort, rectal colonization by MDR bacteria was high (47%; 198 patients), with 131 cases at admission (66.2%) and 67 cases during follow-up (33.8%), with VRE being the most common colonizing microorganism. MDR rectal colonization was also linked to an increased risk of MDR infection, with infections in MDR carriers mainly caused by the colonizing strain [37].

A large study conducted on ICU patients with bacteremia from 24 countries showed that 69.6% of the isolates were recovered from hospital-acquired infections and the most common pathogens were *K. pneumoniae* (23.8%), coagulase-negative staphylococci (17.9%), *A. baumannii* (11%) and *S. aureus* (8.9%). The majority of the isolates were MDR (70%), with *K. pneumoniae* being the most common one among the MDR bacteria (32.1%). The most common resistance profiles were ESBL producers (33.1%), MRSA (7%), CRE (4.2%) and VRE (1.6%), and among the ESBL producers and CRE, the most frequent ones were *K. pneumoniae* and *E. coli* [38]. A multicenter study on carbapenem-resistant *P. aeruginosa* isolated from ICU patients showed that 35% of the strains were carbapenemase-positive. The most common carbapenemase found was VIM (54%), followed by GES (27%), IMP (7%) and NDM (5%) [39].

## 4. Colonization and Infection with MDR Bacteria in HIV-Positive Patients

Given their immunosuppressive status, frequent contact with the medical system and use of antiretrovirals, and the various prophylactic treatments they undergo, HIV-positive patients are more susceptible to infections with antibiotic-resistant bacteria and may develop life-threatening bacteremic infections [40]. The presence of ESBL-producing *Enterobacterales* and MDR *Enterobacterales* is alarming in current practice because it may worsen the prognosis of infectious diseases, especially in HIV-positive individuals [41,42]. Colonization with ESBL-producing *Enterobacterales* frequently precedes invasive infections. Intestinal carriage of ESBL-producing *Enterobacterales* poses a high risk for HIV-positive patients because of the increased risk of developing severe bacterial infections [43,44]. 

Several studies assessed the impact of colonization or infection with MDR bacteria in HIV-positive patients (Table 2). In Tanzania, studies have shown a high frequency of infections caused by ESBL-producing *Enterobacterales* in hospitalized patients. Carriage with ESBL-producing *Enterobacterales* is also common in hospital settings, and a 50% carriage rate has been documented in hospitalized children in Tanzania [45,46]. Another study in Tanzania on 595 newly diagnosed patients with HIV showed that 194 (32.6%) of them were carriers of ESBL-producing *Enterobacterales*. Patients with low CD4+ counts (<350/mL) had a higher colonization rate (44.8%) compared to those with CD4+ counts above 350/mL (31.3%). The majority of patients (74.2%) were colonized with a single bacterial species and 25.8% of patients were colonized with two or more bacterial species. The most common species isolated were *E. coli* (85.7%), followed by *K. pneumoniae* (13.5%) and *E. cloacae* (0.8%). Patients with low CD4+ counts were more likely to be colonized with multiple ESBL-producing *Enterobacterales* compared to patients with high CD4+ counts. Upon genotyping, the majority of strains had *bla*_CTX-M_ genes (97.5%), with the predominance of the *bla*_CTX-M-15_ gene (92%) [47]. Another study conducted in Tanzania on 399 HIV-positive children below 5 years old showed that 27 (6.8%) had nasopharyngeal colonization with *S. aureus,* and 16 (59.5%) of these strains were considered MRSA. The most frequently isolated bacteria from the oral cavity were *K. pneumoniae* (40.7%), *E. coli* (18.5%) and *S. pneumoniae* (14.8%). Colonization with ESBL-producing *Enterobacterales* was present in 92 (33.1%) children, with the most frequently identified species being *E. coli* (63.7%) and *K. pneumoniae* (10.4%). They also showed that GNB isolated from rectal swabs were more resistant to ciprofloxacin, amoxicillin and clavulanic acid, and ceftriaxone compared to GNB isolated from the oral cavity, and also GNB isolated from rectal swabs had a higher ESBL phenotype compared to GNB isolated from the oral cavity. Moreover, bacterial strains isolated from HIV-positive children expressed higher resistance to gentamicin, amoxicillin and clavulanic acid, and meropenem compared to those isolated from HIV-negative children [48]. In a study of 185 patients with HIV in Cameroon, 150 patients (81.08%) were found to be colonized with MDR *Enterobacterales*, of which 61 (40.67%) were colonized with two or more MDR strains. The majority of the strains (66.74%) were ESBL-negative strains and 33.26% were ESBL-positive strains. The most common MDR bacterial species isolated were *E. coli* (78.49%) and *K. pneumoniae* (5.91%). Genotyping of the strains found that *Enterobacterales* showed *bla*_TEM_ (62.9%), *bla*_CTX-M_ (40.86%) and *bla*_SHV_ (10.57%) genes, and in ESBL-positive strains, the most common gene was the *bla*_CTX-M_ gene (70.49%) [49]. In another study conducted in Cameroon on 120 women with HIV, 37 (30.83%) were found to be colonized with *Enterobacterales*. Of the MDR strains, the most frequently isolated were *E. coli* (56%) and *K. pneumoniae* (20%), and 48% of the strains were ESBL-positive. Genotyping confirmed the presence of *bla*_CTX-M_ (48%) and *bla*_TEM_ (72%) genes [50]. In Cameroon, antibiotic resistance of enterobacteria such as *Klebsiella*, *Enterobacter*, *Citrobacter*, *Salmonella* and *Serratia* was significantly higher in HIV-positive compared to HIV-negative patients [51]. In a study from Nepal on HIV-positive patients with lower respiratory tract infections, the most frequently isolated bacteria from these infections were *K. pneumoniae* (25.37%), *H. influenzae* (20.9%) and *E. coli* (17.91%). The majority of bacterial isolates (52.83%) were MDR and 43.39% were ESBL producers. Of those strains that produce ESBL, 47.83% had the *bla*_CTX-M_ gene, 8.6% had the *bla*_TEM_ gene and 43.48% had both genes [52]. A series of studies from Ethiopia focused on colonization and infections with *Enterococcus* spp. In one of the studies, 123 out of 200 patients (61.5%) were colonized with *Enterococcus* spp. Almost half of the strains (49.59%) were considered MDR and 11.4% of the strains were resistant to vancomycin. This study also showed that people with previous exposure to antibiotics for more than two weeks and hospitalization for more than six months had a higher rate of colonization with VRE [53]. In another study, 95 out of 170 patients (56%) were colonized with *Enterococcus* spp. The majority of strains (87.4%) were MDR and 13.7% were resistant to vancomycin. This study demonstrated as well that previous hospitalization and exposure to antibiotics were risk factors for increased colonization with VRE [54]. Another study from Ethiopia showed different results regarding colonization than previous ones. This time, patients included in the study had a lower rate of colonization with *Enterococcus* spp. (8.85%—34 out of 384 patients), but the percentages of resistance were high (MDR—82.35% and VRE—47.05%) as shown in the other two studies [55]. According to a study conducted in the United States from 2000 to 2018, the prevalence of antibiotic resistance of *Enterobacterales* is higher in HIV-positive patients compared to HIV-negative individuals. Of the total number of strains isolated, 16.6% were MDR, and the prevalence was higher in HIV-positive patients (21.5%) compared to HIV-negative patients (16.5%). The most common species isolated were *E. coli* (58.5%), *K. pneumoniae* (15.7%) and *P. mirabilis* (7.5%). An increased rate of resistance to penicillins and combinations of penicillins with beta-lactamase inhibitors was observed in HIV-positive patients. Also, the highest rate of resistance was for sulfonamides, probably also due to the use of a trimethoprim and sulfamethoxazole combination as prophylaxis against *Pneumocystis carinii* infection in patients with a low count of CD4+ [56]. The same authors showed in another study that the presence of MDR *Enterobacterales* was strongly associated with a nadir CD4 cell count ≤ 200 cells/mm^3^, a history of an AIDS-defining clinical condition and hospital admission in the prior 12 months [57].

Several studies assessed the nasal colonization with MRSA in HIV-positive patients. A study from China that included a total of 1001 HIV-positive patients showed that the prevalence of MDR *S. aureus* nasal carriage was 15.18% (152 out of 1001), with 60.08% (152 out of 253) of the *S. aureus* isolates exhibiting multidrug resistance. The majority of MDR *S. aureus* isolates were resistant to three (23.03%, 35 out of 152), four (24.34%, 37 out of 152) or five antimicrobial categories (22.37%, 34 out of 152). Notably, 46 isolates (30.26%, 46 out of 152) displayed resistance to more than six antimicrobial categories. In this study, a history of respiratory tract infections was identified as a risk factor for MDR *S. aureus* nasal carriage [58]. A study from Taiwan on 553 patients showed that only 19 patients (3.4%) were colonized with MRSA, and the risk factors significantly associated with MRSA colonization were cancer and antibiotic use within the past year [59]. A study from Nepal on 400 people (200 HIV-positive people and 200 healthy people) showed a lower prevalence of MRSA nasal colonization—3.5% (7 out of 200) of the HIV-positive cohort and 5% (10 out of 200) of the control cohort. This study found no significant difference in MRSA nasal colonization between HIV-positive people and healthy controls in the study region, but they showed that a longer duration of antiretroviral therapy significantly reduced the risk of MRSA nasal colonization in HIV-positive people [60]. A study from Ghana regarding colonization with *S. aureus* and MRSA in 107 children with HIV and 107 healthy controls showed that the prevalence of *S. aureus* and MRSA carriage among children with HIV was 44.9% (48) and 5.6% (6), respectively, compared to 23.4% (25) and 0.9% (1) in the control group. HIV infection was significantly associated with *S. aureus* colonization (*p* < 0.001) but not with MRSA colonization (*p* = 0.055). This study concluded that HIV infection is a risk factor for *S. aureus* colonization among children, though it may not be a risk factor for MRSA colonization. Additionally, they identified that the absence of colonization with coagulase-negative staphylococci is a risk factor for *S. aureus* colonization, regardless of HIV infection status [61]. A study from Ethiopia on 206 HIV-positive patients showed that the colonization rates for *S. aureus* and MRSA were 61.7% (127/206) and 28.2% (58/206), respectively. In this study, a recent CD4 count below 200 and being on HAART for 5 years or less were strongly associated with *S. aureus* colonization. Additionally, participants on second- or third-line ARV therapy (due to first-line ART failure) showed a statistically significant association with the prevalence of *S. aureus* and MRSA colonization [62].

The prevalence of MDR *Enterobacterales* strains in HIV-positive patients has been associated with HIV-specific factors, such as low CD4+ counts, in association with other risk factors, such as frequent hospitalization, prior exposure to antibiotics and infection-related comorbidities [53,54,57]. CD4+ is a marker of the severity of HIV-associated immunodeficiency, and low CD4+ counts lead to increased mortality and morbidity as well as opportunistic infections. *Enterobacterales* are bacteria that colonize the gut, and HIV infection disrupts the intestinal mucosal epithelium and microbiota, which favors microbial translocation. Therefore, changes in the gut microbiota associated with HIV infection, as well as the inflammatory response, can lead to colonization with MDR *Enterobacterales* and, consequently, to infections with them [63,64]. A study in HIV-positive patients has shown that HIV infection leads to loss of resistance to colonization with exogenous bacteria. HIV infection is associated with an abundance of phylum *Proteobacteria* and a depletion of phylum *Firmicutes*. In particular, *Burkholderia fungorum* and *Bradyrhizobium pachyrhizi* species colonize the duodenum of HIV-positive patients with low CD4+ T-cell counts and are absent in HIV-negative and HIV-positive patients with normal CD4+ counts. Overpopulation with potential opportunistic pathogens such as *Prevotella*, *Fusobacterium* and *Ralstonia* and the depletion of beneficial bacterial genera such as *Lactobacillus* have also been observed in this category of patients [65].

## 5. Colonization and Infection with MDR Bacteria in Cancer Patients

Patients with prolonged immunosuppression such as cancer patients and patients with drug-induced immunosuppression such as transplant patients are more prone to MDR infections. These infections are the main cause of morbidity and mortality in this category of patients and also threaten the chance of graft survival for transplant patients. In liver and kidney transplant patients, the most common infections are with ESBL-producing *Enterobacterales* and CRE [66]. Studies on hematopoietic stem cell transplant patients have shown that colonization with MDR bacteria is a risk factor and it has been stated that stem cell transplantation may be contraindicated or delayed in case of infection with ESBL-producing or carbapenemase-producing *Enterobacterales*, and in patients colonized with these, transplantation should be delayed until decolonization is achieved [67,68]. 

Various studies have evaluated the prevalence of MDR bacteria in cancer patients (Table 3). A study from Iran showed a worrying increase in metallo-beta-lactamase (MBL)-producing strains in patients with cancer. In this study, MBL-positive strains were isolated from patients with skin cancer (21%), leukemia (21%) and prostate cancer (15.8%). All Gram-negative bacilli isolated were multidrug-resistant and 60% of them were resistant to carbapenems [69]. Another study conducted between 2007 and 2017 on 165 oncology patients with invasive infections showed a predominance of Gram-negative bacilli (65%), of which the most frequently involved was *E. coli* (45.6%), followed by *P. aeruginosa* (7.5%) and *A. baumannii* (4%). The majority of the strains (61%) were MDR, and of the *E. coli* strains, 79.6% were ESBL producers [70]. A study conducted in Syria on 163 patients with hematologic malignancies showed that 38 patients (18.9%) were colonized with ESBL-producing *Enterobacterales* and 30 patients (14.92%) were colonized with CRE. Bloodstream infections occurred in 29 cases (14.42%). GNB bacteremia was observed in 8 of the 38 (21.05%) patients colonized with ESBL-producing *Enterobacterales* and 4 of the 30 (13.33%) patients colonized with CRE. Previous quinolone use was identified as the only independent risk factor for intestinal colonization with MDR *Enterobacterales*. No significant link was found between carriage of ESBL-producing *Enterobacterales* or CRE and an increased risk of subsequent bacteremia. Additionally, there were no significant differences between groups receiving modified versus non-modified treatments regarding the duration of hospitalization, antibiotic therapy or 28-day mortality rate [71]. A retrospective study conducted in Tunisia during 2002–2011 regarding MDR on allogeneic stem cells recipients showed an increased incidence of MDR bacteria. Of the 142 *K. pneumoniae* strains, 34.5% were ESBL producers and 11.46% of the 218 *E. coli* strains were found to be ESBL producers. Additionally, 32.8% of the 210 *P. aeruginosa* strains were resistant to imipenem and/or ceftazidime, while 20.75% of the 106 *S. aureus* strains were methicillin-resistant [72]. In China, Zhu et al. showed that 21 out of 320 patients (6.56%) with acute leukemia had intestinal colonization with CRE. The most common strains identified were *K. pneumoniae* (71.43%), followed by *E. coli* (28.57%). Among the 21 patients with CRE colonization, three (14.29%) developed invasive CRE infections. They also showed that age; use of cephalosporins, penicillins and tigecyclines; and hematopoietic stem cell transplantation status were risk factors for CRE colonization [73]. Chen et al. showed that 98 out of 953 patients (10.3%) with hematologic malignancies were colonized with CRE. Of these 98 colonized patients, 18 (18.4%) went on to develop subsequent infections. The majority of the colonizing CRE isolates were *K. pneumoniae* (50%), followed by *E. coli* (27.8%) and *E. cloacae* (9.3%). Regarding the subsequent infections, *K. pneumoniae* was the most common species (55.6%), followed by *E. coli* (33.3%) and other species (11.2%). Patients who developed subsequent infections with CRE had a significantly higher mortality rate (33.3% vs. 2.8%; *p* = 0.001). The use of proton-pump inhibitors and admission to the ICU increased the risk of subsequent CRE infection in patients colonized with CRE with hematologic malignancies [74].

In a study conducted on 73 cancer patients in Greece, the most common MDR bacteria isolated were *K. pneumoniae* (37%), MRSA (24%), *A. baumannii* (21%), *P. aeruginosa* (5%), *E. coli* (4%) and VRE (3%). A total of 10 out of 73 patients (14%) were colonized with MDR bacteria before infection, and 15 patients (20%) were colonized or infected at the time of hospital admission [75]. A study from Germany focusing on colonization and infection with VRE in hematologic and oncologic patients showed that 132 out of 555 patients (23.8%) presented with intestinal colonization with VRE. Of these, 84 patients (63.6%) acquired VRE nosocomially. Overall, four patients (0.7% of all patients and 3% of those with intestinal VRE colonization) developed an infection caused by VRE. All infections were nosocomial, occurring on or after the third day of hospitalization, and each of these patients had intestinal VRE colonization at the time of infection. Among the 132 VRE carriers, 21 had a VRE isolate that was phenotypically resistant to both vancomycin and teicoplanin, while 109 carriers had an isolate resistant to vancomycin but susceptible to teicoplanin. One patient harbored two different VRE strains, one susceptible to teicoplanin and the other resistant [76]. Another study from Germany on 295 patients diagnosed with non-small-cell lung cancer (NSCLC) assessed the colonization with MDR bacteria and the negative impact on survival of these patients. Among the analyzed patients, 24 (8.1%) were colonized with MDR bacteria, while 271 patients (91.9%) were classified as MDR-negative during the screening period. *Enterobacterales* were the most commonly detected MDR bacteria, accounting for 79.2% (19/24) of cases. All of these isolates exhibited phenotypical resistance to third/fourth-generation cephalosporins (ceftriaxone, Cefotaxime, ceftazidime and Cefepime). Additionally, most species were resistant to piperacillin, and more than half showed resistance to trimethoprim/sulfamethoxazole. All the detected MDR *Enterobacterales* were susceptible to carbapenems (imipenem, meropenem and Ertapenem). *E. faecium* resistant to ampicillin, carbapenems and fluoroquinolones (Levofloxacin, ciprofloxacin and Moxifloxacin) was found in 16.7% (4/24) of the MDR-positive cases. Of these, three exhibited the *VanB* phenotype and one the *VanA* phenotype. Additional resistance to high-level aminoglycosides and tetracyclines was detected in one case each. Within the screening period, 25% of the MDR-positive patients became colonized with multiple MDR bacteria; three patients acquired additional ESBL-producing species, and three acquired additional VRE. MDR bacterial colonization was observed in patients across all disease stages and was more prevalent in those with concomitant diabetes mellitus. MDR-positive patients had a significantly higher rate of non-cancer-related mortality compared to MDR-negative patients and a significantly higher rate of mortality due to infectious causes. In this study, MDR bacterial colonization is considered an independent risk factor for impaired overall survival of patients with NSCLC [77]. Another study focused on patients with acute myeloid leukemia undergoing intensive induction chemotherapy. Among 312 patients, 90 were colonized, and 130 were non-colonized. Among the colonized patients, the most commonly detected MDR bacteria was VRE, found in 67 patients (74.4%), followed by 18 ESBL-positive patients (20%) with or without resistance to fluoroquinolones. CRE was identified in 12 patients (13.3%), and MRSA was present in only 2 patients (2.2%). Additionally, nine patients (10%) were colonized by more than one MDR strain. Colonized patients experienced significantly more days with fever, spent more time in the intensive care unit and had higher median C-reactive protein levels during their hospital stay. Despite these findings, there was no prolonged hospital stay or increased mortality rate among colonized patients. However, a subgroup analysis revealed that patients colonized with CRE had significantly lower survival rates at 60 days, 90 days, 1 year and 2 years compared to non-colonized patients [78]. A multicenter cohort study conducted in Italy on patients with hematologic malignancies regarding bloodstream infections with GNB showed that the most frequently isolated species were *E. coli* (440/834, 52.7%), followed by *K. pneumoniae* (160/834, 19.2%), *P. aeruginosa* (122/834, 14.6%), *E. cloacae* (31/834, 3.7%), *A. baumannii* (14/834, 1.7%) and *Stenotrophomonas maltophilia* (14/834, 1.7%). Of the 834 GNB isolates, 256 (30.7%) were classified as MDR. The highest rates of MDR were observed among the *A. baumannii* isolates (64.3%; all of which were XDR) and *K. pneumoniae* isolates (63.1%), followed by *P. aeruginosa* (36.9%), *E. coli* (17.1%) and *E. cloacae* (12.9%). All 14 *S. maltophilia* isolates were considered MDR; of these, 13 out of 14 (92.8%) were susceptible to trimethoprim/sulfamethoxazole. Patients with bloodstream infections with MDR GNB had a longer mean hospital stay and a longer mean time at risk for a bloodstream infection episode compared to those with non-MDR GNB bloodstream infections. Patients with MDR GNB bloodstream infections were more likely to have an indwelling urinary catheter, have undergone total parenteral nutrition, have had previous MDR bacteria culture-positive surveillance rectal swabs, have received antibiotic prophylaxis with fluoroquinolones and have had prior antibiotic therapy, particularly with carbapenems, β-lactams/β-lactamase inhibitors and/or aminoglycosides. Non-MDR GNB bloodstream infections were more common in patients who had undergone autologous HSCT and in those with multiple myeloma. The overall 30-day mortality rate was 16.3% (132/811). Mortality was significantly higher in patients with MDR GNB bloodstream infections compared to those with non-MDR GNB bloodstream infections (88/256, 34.4% vs. 44/555, 7.9%; *p* < 0.001) and in patients who experienced septic shock compared to those who did not (87/181, 48.1% vs. 45/630, 7.1%; *p* < 0.001). MDR GNB bloodstream infections and chronic renal failure were identified as independent risk factors for 30-day mortality, while autologous HSCT was independently associated with improved survival [79]. A study from Spain regarding infections with *P. aeruginosa* in cancer patients showed that diabetes mellitus, previous colonization with MDR *P. aeruginosa*, prior receipt of antibiotics and septic shock acted as risk factors for developing infections with MDR *P. aeruginosa* in immunocompromised patients, who have a poorer outcome than those infected with non-MDR *P. aeruginosa* strains [80]. A prospective study from Turkey regarding rectal colonization with MDR bacteria in 57 patients with hematological malignancies showed that rectal colonization rates were 40.4% (23/57) for ESBL-producing *Enterobacterales* and 8.8% (5/57) for CRE. Bacteremia with ESBL-producing *Enterobacterales* occurred in two (8.6%) of the patients colonized with ESBL-producing *Enterobacterales*, and CRE bacteremia was detected in one (20%) of the patients colonized with CRE. Overall, bacteremia was observed in 24 patients (42.1%), while microbiologically defined infections other than bacteremia were diagnosed in 8 patients (14.1%). Among the bacteremia-causing agents, Gram-positive pathogens were predominant (51.7%). The study also examined risk factors for rectal colonization with ESBL-producing *Enterobacterales* in hematological malignancy patients with febrile neutropenia. Identified risk factors included underlying acute myeloid leukemia; prior chemotherapy; use of beta-lactam antibiotics, quinolones or other antibiotics within the last three months; and recent hospitalization history. A multivariate logistic regression analysis revealed that the use of beta-lactam antibiotics within the last three months increased the risk of rectal colonization with ESBL-producing *Enterobacterales* by 3.5 times (*p* = 0.03) [81].

A study from Brazil that included 714 patients showed that 140 (19.6%) were colonized with carbapenem-resistant GNB or VRE. Colonized patients more often came from the ward, had longer hospital stays before ICU admission, experienced unplanned ICU admissions, had higher predicted mortality at ICU admission and had more hematological malignancies compared to non-colonized patients. None of the colonized patients developed an infection from the same bacteria during their hospital stay, but 20.7% developed such infections after discharge. In-hospital mortality was higher among colonized patients than non-colonized patients (44.3% vs. 33.4%; *p* < 0.01), but in-hospital mortality was not associated with colonization. Patients that were colonized had a poorer health status compared to patients without colonization; however, colonization was not associated with in-hospital mortality and one-year survival [82]. 

A study on 232 hematopoietic stem cell transplant recipients showed that the cumulative incidence of bloodstream infections (BSIs) was 25.4%, with the majority (55.2%) caused by Gram-negative bacteria. Ninety-four patients (40.5%) had intestinal colonization with one or more MDR bacteria at the time of the transplant, including VRE and carbapenem-resistant GNB. Among those colonized by MDR GNB, 20% developed a BSI with the same resistance pattern. Of the thirteen infection-related deaths, ten were in patients with a BSI caused by MDR GNB. Independent risk factors for BSIs included gut colonization by MDR bacteria, particularly GNB and neutropenia lasting longer than 10 days. Risk factors specifically associated with BSIs caused by MDR bacteria were age over 62 years, use of total parenteral nutrition and previous gut colonization by MDR GNB. These findings suggest that previous gut colonization by MDR bacteria is an independent risk factor for BSI and impacts outcomes, as do total parenteral nutrition use and age. Therefore, gut decolonization may be a potential strategy to prevent BSIs [83]. Battipaglia et al. conducted a study in 2019 to evaluate the efficacy of gut microbiota transplantation in eradicating MDR bacteria in patients with pre- or post-hematopoietic stem cell transplant hematologic disease. Patients included in the study were colonized or had infections with carbapenemase-producing GNB or VRE. Decolonization was achieved in 7 out of 10 cases, demonstrating that gut microbiota transplantation is a decolonization strategy that can be safely performed even in patients with hematologic neoplasia colonized with MDR bacteria [84]. According to another study, performed on 120 post hematopoietic stem cell transplant patients, the composition of the gut microbiota may influence immediate complications, such as bacterial infections and acute rejection disease. This study looked at the impact of pre-transplant MDR bacteria colonization and the impact of antibiotic use against anaerobic bacteria. Colonization with MDR bacteria was present in 42.5% of patients, and 32.5% of patients had infections caused by MDR bacteria. Previous colonization was significantly associated with MDR bacterial infections, especially systemic infections. Colonization had a negative impact on post-transplant survival and infection-related mortality [85].

## 6. Research Gaps, Future Directions in Research on Multidrug-Resistant Bacteria Infection versus Colonization in Immunocompromised Patients

There is a need for more precise and standardized diagnostic criteria and methods to distinguish between MDR bacterial colonization and infection in immunocompromised patients, as current diagnostic tools and approaches exhibit significant variability in accuracy and applicability. Additionally, further research is required to understand the implications of MDR bacteria in immunocompromised patients, particularly regarding the interaction between these pathogens and the host immune system, and how this contributes to colonization versus infection. Additional studies are needed to thoroughly analyze the clinical outcomes and prognosis associated with MDR bacterial colonization versus infection in immunocompromised patients, with an emphasis on long-term health impacts and quality of life. Research examining the effect of distinguishing between colonization and infection on antimicrobial stewardship programs is limited, necessitating studies to explore how this distinction influences antibiotic prescribing practices and resistance patterns. Furthermore, there is insufficient evidence regarding the most effective infection control practices and protocols specifically designed for managing MDR bacteria in immunocompromised patients, highlighting the need for comparative studies on various infection control measures. Comprehensive epidemiological data on the prevalence and patterns of MDR bacterial colonization and infection in immunocompromised patients across various regions and healthcare settings are limited, necessitating more large-scale, multicenter studies. Additionally, research is needed to develop and evaluate patient-centered care models that address the specific needs of immunocompromised patients with MDR bacterial colonization or infection, focusing on personalized treatment plans and support systems. Information on the economic impact of managing MDR bacterial colonization versus infection in immunocompromised patients is also limited. Studies should assess the cost-effectiveness of different management strategies and their implications for healthcare systems. Further research is essential to identify and evaluate public health interventions that can effectively reduce the burden of MDR bacteria in immunocompromised populations, including preventive measures and educational programs. Additionally, long-term longitudinal studies are needed to monitor the progression of MDR bacterial colonization and infection in immunocompromised patients, offering insights into chronic outcomes and potential late effects of these conditions.

Future research endeavors should prioritize the development and validation of standardized diagnostic criteria and methodologies aimed at effectively distinguishing between MDR bacterial colonization and infection in immunocompromised cohorts. Such initiatives are aimed to bolster diagnostic consistency and reliability across diverse healthcare contexts. Additionally, delving into the molecular and cellular intricacies governing the interaction between MDR bacteria and the host immune system in immunocompromised individuals holds promise for elucidating the underlying mechanisms of colonization and infection. These insights have the potential to facilitate the improvement of precision-targeted therapeutic modalities. Longitudinal investigations are warranted to evaluate the enduring health ramifications and quality of life among immunocompromised individuals afflicted with either MDR bacterial colonization or infection. Such inquiries should encompass an exploration of the influence of these conditions on morbidity, mortality and overall patient prognostication. Furthermore, additional research endeavors should scrutinize the repercussions of discerning between colonization and infection on antimicrobial stewardship programs, including an assessment of its implications for antibiotic prescribing practices, resistance patterns and clinical outcomes. Essential to this pursuit is comparative inquiry into diverse infection control practices and protocols tailored specifically for the management of MDR bacteria in immunocompromised populations. The identification of optimal strategies in this regard holds the potential to mitigate the dissemination of these pathogens within healthcare facilities. Significant multicenter epidemiological studies of considerable scope are imperative to furnish substantial data pertaining to the prevalence, patterns and trends characterizing MDR bacterial colonization and infection in immunocompromised cohorts, spanning diverse geographic regions and healthcare contexts. Additionally, research endeavors should incorporate comprehensive economic evaluations of varied management strategies employed for addressing MDR bacterial colonization and infection. A nuanced understanding of the cost-effectiveness inherent in these strategies is set to inform judicious resource allocation and facilitate the development of informed policy frameworks. Subsequent research endeavors should prioritize the development and evaluation of patient-centered care models tailored specifically to address the distinctive needs of immunocompromised individuals contending with MDR bacterial colonization or infection. These models should underscore the implementation of personalized treatment regimens and comprehensive support frameworks. The identification and assessment of efficacious public health interventions aimed at alleviating the burden of MDR bacteria in immunocompromised populations constitute a pivotal area of research. Accordingly, research initiatives should explore a spectrum of preventive measures, educational initiatives and policy interventions aimed at ameliorating the impact of these infections. Moreover, conducting extended longitudinal studies is crucial for gaining insights into the long-term outcomes and potential delayed effects of MDR bacterial colonization and infection. Simultaneously, interventional studies offer significant potential for assessing new therapeutic approaches, preventive measures and management strategies aimed at improving patient outcomes.

## 7. Conclusions

This review assessed the relations between colonization and infection with MDR bacteria in immunocompromised patients, such as ICU patients, HIV-positive patients and cancer patients. Some studies focused only on colonization or infection with MDR bacteria without assessing how both can influence each other, while some studies focused on both, emphasizing the impact that colonization with such pathogens can have on the outcome of this vulnerable category of patients. In the majority of studies, there was a high prevalence of *Enterobacterales*, especially ESBL producers, with *E. coli* and *K. pneumoniae* being frequently present across multiple studies, followed by non-fermenting GNB, such as *A. baumannii* and *P. aeruginosa*. Most strains of *A. baumannii* and *P. aeruginosa* had increased rates of resistance to carbapenems. So, these studies showed that MDR GNB represent the main threat to immunocompromised patients, which can produce infections that are difficult to treat given the fact that there is a lack of active antibiotics against this category of pathogens, and in most circumstances, infections with MDR GNB have a negative impact on the clinical outcome and the quality of life of immunocompromised patients. On the other hand, regarding Gram-positive bacteria, a growing threat is represented by vancomycin-resistant *Enterococcus* spp., which was highlighted in different studies, as well as MRSA. Studies across different regions of the world showed a variety of patterns regarding the prevalence of MDR bacteria, which shows that the epidemiological distribution of MDR bacteria demonstrates significant regional variability, reflecting the influence of diverse factors such as healthcare practices, antibiotic usage patterns and local microbial ecology across different geographical areas. While in some cases colonization with MDR bacteria proved to be an important risk factor for MDR bacterial infections, some failed to assess this risk, so further research in this area is needed. The most frequently encountered risk factors associated with colonization or infection with MDR bacteria were previous exposure to antibiotics, increased hospital stay or previous hospitalization and the use of invasive medical equipment. Additional studies are needed to thoroughly analyze the clinical outcomes and prognosis associated with MDR bacterial colonization versus infection in immunocompromised patients, with an emphasis on long-term health impacts and quality of life. Comprehensive epidemiological data on the prevalence and patterns of MDR bacterial colonization and infection in immunocompromised patients across various regions and healthcare settings are limited, necessitating more large-scale, multicenter studies. Additionally, research is needed to develop and evaluate patient-centered care models that address the specific needs of immunocompromised patients with MDR bacterial colonization or infection, focusing on personalized treatment plans and support systems. Further research is essential to identify and evaluate public health interventions that can effectively reduce the burden of MDR bacteria in immunocompromised populations, including preventive measures and educational programs. Long-term longitudinal studies are needed to monitor the progression of MDR bacterial colonization and infection in immunocompromised patients, offering insights into chronic outcomes and the potential late effects of these conditions.

## Figures and Tables

**Table 1:** Prevalence of MDR bacteria involved in colonization/infection of ICU patients and risk factors associated with colonization/infection.

Group Study	Colonization with MDR Bacteria	Infection with MDR Bacteria	Resistance Pattern	Risk Factors for Colonization/Infection	Study
ICU patients	*E. coli* (57%) *K. pneumoniae* (16%) *C. freundii* (7%) *E. cloacae* (6%) *K. aerogenes* (5%)	-	ESBL-producing *E. coli* and other ESBL producers (76%) Derepressed ampC producers (15%)	Liver cirrhosis Previous MDR-GNB carriage Digestive surgery in the last year Length of hospital stay	Fernandez-Martinez et al. (2022) [21]
ICU patients	VRE (16%) *K. pneumoniae* (3%)	-	*VanA* (81%) OXA-48 NDM	-	Karașin et al. (2021) [22]
ICU patients	-	*A. baumannii* (35.5%) *K. pneumoniae* (20.6%) *Enterococcus* spp. (13%) *P. aeruginosa* (11.5%) *S. aureus* (8%) *E. coli* (6.9%)	MDR (12.5%) XDR (49.2%) PDR (1.2%)	-	Routsi et al. (2020) [23]
ICU patients	39.04% colonized with Gram-negative bacteria 7.61% colonized with Gram-positive bacteria 2.61% colonized with both	45.20% with MDR colonization developed sepsis 32.07% with XDR colonization developed an infection	MDR (43.71%) XDR (31.73%)	ICU length of stay Use of external ventricular drainage and intracerebral pressure-monitoring catheters	Munari et al. (2022) [24]
ICU patients	51.85% colonized by carbapenem-resistant *A. baumannii* and/or carbapenem-resistant *K. pneumoniae*	-	Carbapenem resistance	Longer ICU stay Extended periods of mechanical ventilation Strong statistical association between prior colonization and the subsequent development of infection	Ceccarelli et al. (2022) [25]
ICU patients	68.8% colonized with one bacterial strain—*E. coli*, *Klebsiella* spp., *S. aureus* and *E. faecium* 24.5% colonized with 2 different bacterial strains—*Klebsiella* spp. and *E. faecium* 5.7% colonized with 3 different bacterial strains	-	ESBL producers (75.5%) Carbapenemase producers (32.1%) MRSA (11.3%) VRE (3.8%)	Carmeli score = 3 Previous exposure to antibiotics and hospitalization in the past 6 months	Vlad et al. (2023) [26]
ICU patients	28%—carbapenem-resistant *K. pneumoniae*	45.9%—subsequent infection with carbapenem-resistant *K. pneumoniae*	Carbapenem resistance	-	Qin et al. (2020) [27]
ICU patients	Carbapenemase-producing *Enterobacterales * 66.2% were colonized upon ICU admission 33.8% acquired them during their ICU stay	-	Carbapenemase producers 89.6% KPC	ICU length of stay Open wounds The presence of catheters or tubes Antibiotic treatment	Kim et al. (2023) [28]
ICU patients	-	*K. pneumoniae* (21%) *P. aeruginosa* (11.8%) *S. aureus* (13.2%)	85.1% MDR 28.6% XDR 4.6% PDR	Advanced age (>75 years old) Male gender	Wani et al. (2021) [29]
Neonatal ICU patients	43% colonized with *K. pneumoniae*, *Acinetobacter* spp., *E. coli* and *C. freundii*	Bacteremia (34.5%) with GNB (85.5%)	Resistance to third-generation cephalosporins	Cyanosis, jaundice, number of invasive devices and contaminated cots were associated with GNB bacteremia	Silago et al. (2020) [30]
ICU patients	54.54%—fecal carriage of ESBL-producing *Enterobacterales* 9.32%—nasal carriage of MRSA	-	ESBL producers MRSA	Hospitalization for two or more days before ICU admission Antibiotic use prior to ICU admission (not statistically significant)	Manyahi et al. (2022) [31]
ICU patients	-	56%—GNB infections *E. coli* (33.3%) *K. pneumoniae* (31.1%) *P. aeruginosa* (14.4%)	92% MDR Third-generation cephalosporins resistance Carbapenem resistance Colistin resistance	Previous use of antibiotics Use of nasogastric tube Respiratory and cardiovascular-associated conditions	Maina et al. (2023) [32]
ICU patients	-	*A. baumannii* (25.4%) *Acinetobacter* spp. (14.3%) *K. pneumoniae* (13.2%) *P. aeruginosa* (9.6%) *S. aureus* (8.7%)	Carbapenem resistance—all strains of *Acinetobacter* and *P. aeruginosa* MRSA (100%) *K. pneumoniae*—76% ESBL and 22% KPC	Advanced age Use of orotracheal tube High blood pressure, cardiac and pulmonary diseases, chronic kidney disease	Martins et al. (2024) [33]
ICU patients	-	62% had at least one MDR isolate: carbapenem-resistant *A. baumannii* and *P. aeruginosa*, carbapenem-resistant *K. pneumoniae*, MRSA and other carbapenem-resistant *Enterobacterales*	Carbapenem resistance MRSA	-	Oliveira et al. (2023) [34]
ICU patients	-	*P. aeruginosa* (30.17%) *K. pneumoniae* (20.12%) *E. coli* (16.05%)	ESBL producers Carbapenemase producers	-	Uc-Cachón et al. (2019) [35]
ICU patients	33% were colonized with at least one MDR bacteria: 28% were colonized with VRE 6% were colonized with MRSA 3% were colonized with ESBL-producing *Enterobacterales*	Infections occurred in 58% of patients, with 42% of these cases being ICU-acquired, and 12% developed infections with MDR bacteria	ESBL producers VRE MRSA	-	Kim et al. (2023) [36]
ICU patients (Barcelona cohort + Frankfurt cohort)	Barcelona cohort—32.7% were colonized by MDR bacteria, with 64.2% at admission and 35.8% during follow-up Frankfurt cohort—47% were colonized by MDR bacteria, with 66.2% at admission and 33.8% during follow-up	-	Barcelona cohort—ESBL-producing *Enterobacterales* Frankfurt cohort—VRE	-	Prado et al. (2022) [37]
ICU patients	-	69.6%—hospital-acquired infections (bacteremia) *K. pneumoniae* (23.8%) coagulase-negative staphylococci (17.9%) *A. baumannii* (11%) *S. aureus* (8.9%)	70% MDR ESBL producers (33.1%) MRSA (7%) CRE (4.2%) VRE (1.6%)	-	El-Sokkary et al. (2021) [38]
ICU patients	-	*P. aeruginosa*	Carbapenemase producers (35%): VIM (54%) GES (27%) IMP (7%) NDM (5%)	-	Gill et al. (2022) [39]

**Table 2:** Prevalence of MDR bacteria involved in colonization/infection of HIV-positive patients and risk factors associated with colonization/infection.

Group Study	Colonization with MDR Bacteria	Infection with MDR Bacteria	Resistance Pattern	Risk Factors for Colonization/Infection	Study
HIV-positive patients	32.6% colonized with ESBL-producing *Enterobacterales*: *E. coli* (85.7%) *K. pneumoniae* (13.5%) *E. cloacae* (0.8%)	-	ESBL producers 97.5%—*bla*_CTX-M_ genes (92%—*bla*_CTX-M-15_ gene)	Low CD4+ count (<350/mL)	Manyahi et al. (2020) [47]
HIV-positive patients	6.8% colonized with *S. aureus* 33.1% colonized with ESBL-producing *Enterobacterales*: *E. coli* (63.7%) *K. pneumoniae* (10.4%)	-	MRSA (59.5%) ESBL producers	History of antibiotic use in the last month	Msanga et al. (2022) [48]
HIV-positive patients	81.08% colonized with MDR *Enterobacterales*, of which 40.67% were colonized with two or more MDR strains *E. coli* (78.49%) *K. pneumoniae* (5.91%)	-	ESBL producers (33.26%) *bla*_TEM_ (62.9%) *bla*_CTX-M_ (40.86%) *bla*_SHV_ (10.57%)	-	Dimani et al. (2023) [49]
HIV-positive patients	30.83% colonized with *Enterobacterales*: *E. coli* (56%) *K. pneumoniae* (20%)	-	ESBL producers (48%) *bla*_CTX-M_ gene (48%) *bla*_TEM_ gene (72%)	-	Zemtsa et al. (2022) [50]
HIV-positive patients	-	Lower respiratory tract infections: *K. pneumoniae* (25.37%) *H. influenzae* (20.9%) *E. coli* (17.91%)	ESBL producers (43.39%) *bla*_CTX-M_ gene (47.83%) *bla*_TEM_ gene (8.6%) both genes (43.48%)	CD4+ count < 200 cells/μL	Maharjan et al. (2022) [52]
HIV-positive patients	61.5% colonized with *Enterococcus* spp.	-	MDR (49.59%) VRE (11.4%)	Previous exposure to antibiotics for more than two weeks Hospitalization for more than six months	Regasa et al. (2021) [53]
HIV-positive patients	56% colonized with *Enterococcus* spp.	-	MDR (87.4%) VRE (13.7%)	Previous exposure to antibiotics Previous hospitalization	Zike et al. (2024) [54]
HIV-positive patients	8.85% colonized with *Enterococcus* spp.	-	MDR (82.35%) VRE (47.05%)	Previous exposure to antibiotics Previous hospitalization	Tilahun et al. (2023) [55]
HIV-positive patients	-	The prevalence of MDR bacteria was higher in HIV-positive patients (21.5%) compared to HIV-negative patients (16.5%). The most common species isolated were *E. coli* (58.5%), *K. pneumoniae* (15.7%) and *P. mirabilis* (7.5%)	MDR (16.6%) Increased rate of resistance to penicillins, combinations of penicillins with beta-lactamase inhibitors and sulfonamides	-	Henderson et al. (2022) [56]
HIV-positive patients	-	1.6%—infections with MDR *Enterobacterales*	MDR	Nadir CD4 cell count ≤ 200 cells/mm^3^ A history of an AIDS-defining clinical condition Hospital admission in the prior 12 months	Henderson et al. (2022) [57]
HIV-positive patients	*S. aureus* nasal carriage was 15.18% with 60.08% of the *S. aureus* isolates exhibiting multidrug resistance	-	MRSA	A history of respiratory tract infections	He et al. (2021) [58]
HIV-positive patients	3.4% colonized with MRSA	-	MRSA	Cancer Antibiotic use within the past year	Hsu et al. (2020) [59]
HIV-positive patients	Lower prevalence of MRSA nasal colonization—3.5% (7 out of 200) of the HIV-positive cohort and 5% (10 out of 200) of the control cohort	-	MRSA	No significant difference in MRSA nasal colonization between HIV-positive people and healthy controls	Kapali et al. (2021) [60]
HIV-positive patients	Prevalence of *S. aureus* and MRSA carriage among children with HIV was 44.9% and 5.6%, respectively, compared to 23.4% and 0.9% in the control group	-	MRSA	HIV infection is a risk factor for *S. aureus* colonization among children, though it may not be a risk factor for MRSA colonization The absence of colonization with coagulase-negative staphylococci is a risk factor for *S. aureus* colonization	Donkor et al. (2019) [61]
HIV-positive patients	Colonization rates for *S. aureus* and MRSA were 61.7% and 28.2%, respectively	-	MRSA	CD4 count below 200 Being on HAART for 5 years or less	Muhaba et al. (2022) [62]

**Table 3:** Prevalence of MDR bacteria involved in colonization/infection of cancer patients.

Group Study	Colonization with MDR Bacteria	Infection with MDR Bacteria	Resistance Pattern	Risk Factors for Colonization/Infection	Study
Cancer patients	-	*E. coli* (71.8%) *Klebsiella* spp. (22.3%) *Proteus* spp. (2.9%)	All isolates were MDR and 60% of them were carbapenem-resistant 31.7% were MBL-positive	-	Zare et al. (2019) [69]
Cancer patients	-	65% invasive infections with GNB *E. coli* (45.6%) *P. aeruginosa* (7.5%) *A. baumannii* (4%)	ESBL producers (79.6%)	-	Haddad et al. (2021) [70]
Cancer patients	18.9% were colonized with ESBL-producing *Enterobacterales* 14.92% were colonized with carbapenem-resistant *Enterobacterales*	Bloodstream infections occurred in 29 cases (14.42%) GNB bacteremia was observed in 8 of the 38 (21.05%) patients colonized with ESBL-producing *Enterobacterales* and 4 of the 30 (13.33%) patients colonized with carbapenem-resistant *Enterobacterales*	ESBL producers Carbapenem resistance	Previous use of quinolones was identified as the only independent risk factor for intestinal colonization with MDR *Enterobacterales*	Alrstom et al. (2021) [71]
Cancer patients	-	34.5%*—K. pneumoniae* ESBL producers 11.46%—*E. coli* ESBL producers 32.8%—*P. aeruginosa* resistant to imipenem and/or ceftazidime 20.75%—MRSA	ESBL producers MRSA Carbapenem resistance	-	Mechergui et al. (2019) [72]
Cancer patients	6.56%—intestinal colonization with carbapenem-resistant *Enterobacterales* 71.43%—*K. pneumoniae* 28.57%—*E. coli*	14.29% developed invasive infections with carbapenem-resistant *Enterobacterales*	Carbapenem resistance	Age Use of cephalosporins, penicillins, tigecyclines Hematopoietic stem cell transplantation status	Zhu et al. (2022) [73]
Cancer patients	10.3% were colonized with carbapenem-resistant *Enterobacterales* 50%—*K. pneumoniae* 27.8%*—E. coli* 9.3%—*E. cloacae*	18.4% developed subsequent infections 55.6%*—K. pneumoniae* 33.3%—*E. coli* 11.2%—other species	Carbapenem resistance	Use of proton-pump inhibitors and admission to the ICU increased the risk of subsequent CRE infection in patients colonized with CRE with hematologic malignancies	Chen et al. (2023) [74]
Cancer patients	14% of patients were colonized with MDR bacteria before infection 20% were colonized upon hospital admission	*K. pneumoniae* (37%) MRSA (24%) *A. baumannii* (21%) *P. aeruginosa* (5%) *E. coli* (4%) VRE (3%)	ESBL producers Carbapenem resistance MRSA VRE	-	Perdikouri et al. (2019) [75]
Cancer patients	23.8% were colonized with VRE	3% of those with intestinal VRE colonization developed an infection caused by VRE	VRE	-	Chhatwal et al. (2020) [76]
Cancer patients	8.1% were colonized with MDR bacteria 79.2% *Enterobacterales* 16.7% VRE	-	ESBL producers VRE	MDR bacterial colonization is considered an independent risk factor for impaired overall survival of patients with NSCLC	Stratmann et al. (2020) [77]
Cancer patients	74.4%—VRE 20%—ESBL-producing *Enterobacterales* 13.3%—Carbapenem-resistant *Enterobacterales* 2.2%—MRSA	-	ESBL producers Carbapenem resistance VRE MRSA	Colonized patients experienced significantly more days with fever, spent more time in the intensive care unit and had higher median C-reactive protein levels during their hospital stay	Ballo et al. (2019) [78]
Cancer patients	-	Bloodstream infections with GNB *E. coli* (52.7%) *K. pneumoniae* (19.2%) *P. aeruginosa* (14.6%) *E. cloacae* (3.7%) *A. baumannii* (1.7%) *Stenotrophomonas maltophilia* (1.7%)	30.7% MDR	MDR GNB bloodstream infections and chronic renal failure were identified as independent risk factors for 30-day mortality	Trecarichi et al. (2023) [79]
Cancer patients	*P. aeruginosa*	*P. aeruginosa*	MDR	Diabetes mellitus Previous colonization with MDR *P. aeruginosa* Prior receipt of antibiotics Septic shock These acted as risk factors for developing infections with MDR *P. aeruginosa*	Hernández-Jiménez et al. (2022) [80]
Cancer patients	40.4% were colonized with ESBL-producing *Enterobacterales* 8.8% were colonized with CRE	Bacteremia with ESBL-producing *Enterobacterales* occurred in 2 (8.6%) of the patients colonized with ESBL-producing *Enterobacterales*, and CRE bacteremia was detected in 1 (20%) of the patients colonized with CRE	ESBL producers Carbapenem resistance	Underlying acute myeloid leukemia Prior chemotherapy Use of beta-lactam antibiotics, quinolones or other antibiotics within the last three months Recent hospitalization history	Kömürcü et al. (2020) [81]
Cancer patients	19.6% were colonized with carbapenem-resistant GNB or VRE	-	Carbapenem resistance VRE	-	Junior et al. (2023) [82]

## Data Availability

The data are contained within the article.
